# Psychosocial Needs of Children in Foster Care and the Impact of Sexual Abuse

**DOI:** 10.1007/s10826-017-0970-7

**Published:** 2017-11-30

**Authors:** Anne Steenbakkers, Ingunn T. Ellingsen, Steffie van der Steen, Hans Grietens

**Affiliations:** 1 Department of Special Needs Education and Youth Care, University of Groningen, Groningen, The Netherlands; 2 Department of Social Studies, University of Stavanger, Stavanger, Norway

**Keywords:** Foster care, Child sexual abuse, Child maltreatment, Subjectivity, Voices of children, Q sort

## Abstract

Children in family foster care, especially those who have experienced sexual abuse, require a safe and nurturing environment in which their psychosocial needs are met. However, there is limited knowledge on how youth prioritize various needs and what impact previous experiences have on these needs. In this study, we asked youth (formerly) in family foster care to indicate their psychosocial needs, and analyzed if youth with a history of sexual abuse have different needs. A Q methodological study was conducted with 44 youth (age 16–28). Fifteen of them reported sexual abuse during their childhood. Using by-person factor analyses, respondents who share similar subjective views were grouped together. Qualitative interpretations of the factors show differences and similarities between and within the two groups, related to help from others, being independent, processing the past, and working toward the future. Although the needs of youth with and without experiences of sexual abuse seem mostly similar, one group of sexually abused youth specifically indicated not wanting an emotional connection to foster parents, but instead a strictly instrumental, professional relationship. This study captured the diverse perspectives of youth themselves, revealing that children in foster care differ with regard to what they consider as (most) important safety, belonging, self-esteem and self-actualization needs.

## Introduction

Children in family foster care are often seen as a highly vulnerable group, since they have encountered many adverse experiences before placement (Greeson et al. 2011). The foster care system aims to provide a safe and nurturing environment that meets the needs of children and youth so they can thrive (e.g., Leslie et al. 2005). Cooperation between foster parents, birth parents and professionals is vital for successful family foster care placements, as well as prioritizing permanency for children and addressing any delays they might have (Leslie et al. 2005; Pasztor et al. 2006). However, both during foster care and afterward, these children seem to fare worse than other children.

Research suggests that children in foster care have high rates of externalizing and internalizing behavioral problems (see Oswald et al. 2010 for a review), which do not seem to decrease during their stay in foster care (see Goemans et al. 2015 for a meta-analysis). While the cognitive, adaptive and behavioral functioning of children in foster care is worse compared to the general population and less severe compared to children in more restrictive out-of-home care settings (Goemans et al. 2016; Leloux-Opmeer et al. 2017), the outcomes of children in foster care do not seem to differ from at-risk youth who remained at home (Goemans et al. 2016). After leaving care, young adults formerly in foster care more often have fragile economic positions (Pecora et al. 2006), difficulties finding emotional and practical support (Hiles et al. 2014), and fewer of them complete post-secondary education (Pecora et al. 2006). These studies indicate that there is room for progress in the efforts of foster care professionals to accommodate and meet the psychosocial needs of children.

Children in foster care who have experienced sexual abuse seem specifically at risk. Studies have shown that they, to a greater extent than other youth, are exposed to more types of maltreatment, display more behavioral problems, show higher risk of dropping out of school, and are more often incarcerated (Edmond et al. 2002). Moreover, they experience repeated out-of-home placements (Eggertsen 2008; Pollock and Farmer 2005), have a higher rate of prior family dysfunction (Pollock and Farmer 2005), and are more often diagnosed with post-traumatic stress disorder (Dubner and Motta 1999). Many of these children need to learn how to give and receive affection appropriately and how to manage personal boundaries (Farmer and Pollock 2003). These youth need foster parents who are particularly secure and trauma sensitive, and some may need additional therapeutic interventions (Pollock and Farmer 2005). Foster parents are rarely aware of the history of sexual abuse, making it more difficult to meet the specific needs of this group of children (Pollock and Farmer 2005).

Accurately meeting the psychosocial needs of children in foster care may lead to more stable and secure placements, which can help them overcome previous adversities and prepare for adult life (Berrick and Skivenes 2012; Pollock and Farmer 2005). People have five basic needs that promote a healthy development, presented by Maslow (1943) as a hierarchy in which lower order needs have to be satisfied first: physiological needs, the need for safety, the need to belong, the need for self-esteem and the need for self-actualization. The last four of Maslow’s needs are considered psychosocial needs and are subdivided in ‘deficiency needs’ (safety and belonging) and ‘growth needs’ (self-esteem and self-actualization) (Maslow 1954). Research has shown that meeting deficiency needs positively influences growth needs (Noltemeyer et al. 2012).

Research has mainly focused on a single subset of needs, such as belonging (e.g., Buehler et al. 2006; Schofield and Beek 2009). However, as Maslow’s (1943) theory highlights, the four psychosocial needs influence one another and should thus also be studied simultaneously. It is important to ask youth about these needs, because they can best indicate what needs are most urgent for them (Mason 2008; McGregor et al. 2009). In addition, research has not focused on how needs differ across groups of children with diverse previous experiences. As argued, children in foster care with a history of sexual abuse may have specific needs, which are possibly not as urgent for non-sexually abused children (Farmer and Pollock 2003; Pollock and Farmer 2005).

In the Netherlands, where this study is situated, the majority of children who are placed in out-of-home care are placed with a foster family. The permanency aim for children who continue to require out-of-home services is to remain in a stable foster family (De Baat et al. 2017). In order to stimulate children’s well-being and permanency, it is important to study what children and young adults themselves indicate as their most important needs. By using a Q methodological approach (Stephenson 1953), this study illustrates different viewpoints of groups of youth regarding their own psychosocial needs. The aim of this study is twofold. First, we want to describe the psychosocial needs among youth in family foster care who *do* and *do not* report sexual abuse. Secondly, we want to assess the differences and similarities in the needs of these two groups. These aims lead to two main research questions. The first research question is: ‘What are the psychosocial needs of children in foster care according to their own views?’ The second question is: ‘What is the influence of a self-reported history of sexual abuse on these needs?’ In this study, we define the term “need” as a motivating force that directs the behavior, thoughts and emotions of a person (Ryan 1995). Sexual abuse is defined as ‘the involvement of dependent, developmentally immature children and adolescents in sexual activities that they do not fully comprehend, to which they are unable to give informed consent, or that violate the social taboos of family roles’ (Kempe 1977, p. 382). A broad range of sexual activities fall under this definition, ranging from intercourse and attempted intercourse, to oral-genital contact, fondling of genitals directly or through clothing, exhibitionism or exposing children to adult sexual activity or pornography, and the use of children for prostitution or pornography (Putnam 2003).

## Method

### Participants

The participants of this study were a purposive sample of older adolescents and young adults. We believed older children in family foster care and young care leavers would have more recent placement memories and be able to reflect on their memories both in a present and a retrospective perspective. A second criterion was that participants had lived with one foster family for at least one year, which increased the likelihood for them to bond with their foster parents. The final sample consisted of 44 Dutch adolescents and young adults (formerly) living in a foster family. There were 35 women and nine men who on average were 20.95 years old (SD = 2.95, range 16–28). The majority reported a Dutch ethnicity (*n* = 36), while a subset reported a Dutch Antilles, Surinam, Egyptian, Hindu or English background. Their foster care experiences varied in terms of number of foster families they had lived with (range 1–9, M = 2.36), their longest stay with one family (range 1–20 years, M = 7.86), and the age they were placed into care (range 4 months to 17 years, M = 8.35 years). At the time of the study, most participants lived independently (64%), almost one-third lived with their foster family, and two lived with their birth parents.

### Procedure

The aim of a Q methodological study is to reveal patterns of subjectivity, such as views, beliefs and opinions, among participants, allowing researchers to see certain issues from the participants’ point of view (McKeown and Thomas 1988). Moreover, Q methodology is well-suited for research with youth and vulnerable groups, because participants do not necessarily have to disclose their thoughts verbally (Ellingsen et al. 2010). In Q methodological studies, participants rank a set of statement cards about the topic of interest (the so-called Q-sort), revealing how they identify with these statements (Watts and Stenner 2012). Q methodology can be considered as a qualitative method applying quantitative techniques (Shemmings and Ellingsen 2012). The methodology illustrates different viewpoints held by groups of participants about the research topic (McKeown and Thomas 1988), in this case, their psychosocial needs.

Written information about the study was provided to potential participants by four foster care organizations, one youth group and one foster parent group. Letters were sent to adolescents currently in care, young adults formerly in care and foster families who had previously cared for youth now emancipated. Youth who were interested in participating were requested to contact the researchers directly. This method limited the possibility to understand why youth did not want to participate, but some reactions we received stated a lack of time, finding it too difficult to participate emotionally, and not wanting to be associated with the stigmatizing label ‘foster child’. Lastly, snowball-sampling was used, by asking participants if they knew other youth (formerly) in family foster care who might be interested in participating. Before the study, participants received thorough information about the research project, and any questions they had were clarified. Informed consent was obtained and all participants agreed to have the interview audio recorded.

#### Ethical considerations

Out-of-home placement and sexual abuse are sensitive issues, requiring particular ethical awareness from the researcher. Hence, participants could decide on the location of the session. They were assured that they did not need to answer questions that made them feel uncomfortable, and that they could take part in the study without any verbal elaborations if they wished so. Furthermore, no inquiries into abuse history were made other than the ACE questionnaire. Participants received the contact information of the researcher in case they had any further questions, as well as the contact details of two independent organizations that could provide after-care if needed. The research procedure was approved by the Ethics Committee of the host institution.

### Measures

#### Questionnaires

We asked participants about their age, ethnicity, foster care experiences and current living situation. In addition, participants reported on their current psychological functioning in the Brief Symptom Inventory (BSI, Derogatis 1975). To measure the currently experienced trauma symptoms, participants filled out the Davidson Trauma Scale (DTS, Davidson 2002). Both questionnaires resulted in a total score, in which a high score indicated more problems.

We also presented participants with the Dutch translation of the Adverse Childhood Experiences (ACE) questionnaire (Felitti et al. 1998). Ten adverse childhood experiences were included in the questionnaire and participants were asked to indicate for each ACE whether they experienced it. ACEs range from physical and sexual abuse to neglect and witnessing domestic violence. For the purpose of this study, the participants were asked if they had these experiences *prior* to foster care, with the exception of sexual abuse that could have occurred anytime during their childhood. The nine questions concerning experiences prior to foster care were summed, resulting in a score between zero and nine ACEs. In addition, participants reported whether an adult or peer had ever, without the participant’s permission, sexually touched them or had the participant sexually touch that person, made or showed sexual images or movies, or had (tried to have) oral, vaginal or anal sex. If any of these experiences were reported, the participant was categorized in the sexual abuse (SA) group. If none of these experiences were reported, the participant was categorized in the no sexual abuse (No SA) group. In total, 15 participants reported experiencing sexual abuse and 29 participants did not report sexual abuse. The age of the participants at the onset of the sexual abuse ranged from 3 to 16 years old (M = 7.47, SD = 3.82).

#### Q Sort

The statement cards used for the Q sort were constructed by conducting episodic interviews (Flick 1997) with 15 youth (formerly) in family foster care (seven reporting to have experienced sexual abuse) about their needs, and seven foster care workers about the needs of children with and without sexual abuse experiences in foster care. Interview fragments that expressed an opinion or experience about one of the four psychosocial needs were identified. In order to reduce the number of statements to a manageable sample for Q sorting, a design inspired by Fisher’s balanced block (Fisher 1960) was applied. This procedure resulted in a set of 80 statements that represented the needs that emerged from the episodic interviews, covering both deficiency and growth needs in relation to key figures in children’s lives (foster parents, birth parents, friends, professionals and themselves). The final set of statements was selected by a focus group consisting of six participants of the episodic interviews and a researcher, resulting in a set of 45 statements. Examples of belongingness statements are: ‘I want my foster family to be a warm home’ and ‘I find it important to tell my birth parents everything about what I am experiencing in my foster family’. The statements were constructed in Dutch and back-translated to English by two independent researchers familiar with the social work field (see Table 1 for the complete list of statements).

**Table 1:** Weighted average Q sorts for the factors of the SA group and the No SA group

	SA group				No SA group			
	1	2	3	4	A	B	C	D
1. I want to be able to be honest about my feelings and to tell my foster parents everything	7	4	5	6	8	**6**	**2**	8
2. I want to have a nice time with my biological parents during contact visits	**3**	6	4	5	2	**8**	3	3
3. I find it important that my friends know that I’ve had a difficult past	3	2	3	1	3	2	**8**	2
4. I want to decide for myself when I have physical contact with someone	8	**4**	7	6	6	6	6	6
5. I want to do everything I can to prevent adopting the negative characteristics of my biological parents	5	5	**9**	4	**9**	3	4	2
6. I want my foster parents to be more preoccupied with my abilities than with my problems	**7**	4	4	5	4	6	6	5
7. I find it important that I have the opportunity to talk to someone who is neutral with respect to the whole situation	4	3	**6**	2	4	5	5	**7**
8. I want to be just as care-free as other youth my age	7	8	4	6	**7**	4	5	4
9. I find it important to search for the role that my biological parents play in my life	3	6	8	4	6	2	4	4
10. I want to be able to take time and space to think about and process my past	7	**1**	6	5	3	3	**7**	4
11. I want my foster family to be a warm home	**8**	9	9	**4**	**7**	9	**4**	9
12. I find it important that others do not start talking about my past without a reason	3	5	3	5	3	2	**6**	3
13. I want to be able to completely trust my guardian, and I want him/her to honor the agreements that are made	5	5	4	**9**	4	4	4	**7**
14. I want my foster parents to stimulate me to do the best I can do at school	5	8	**5**	7	5	6	**1**	4
15. I find it important that the visitation rules with my biological parents are well organized and in accordance with my preferences	4	3	5	4	6	5	**1**	6
16. I want the foster care worker to take conflicts seriously and to mediate when there are conflicts	4	6	8	7	**4**	6	8	6
17. I find contact with pets important because it helps me get through difficult days	4	**7**	**1**	4	2	4	3	1
18. I want to have a lot of room to make my own choices and to become who I want to be	9	9	8	7	9	9	8	**6**
19. I find it important that there is room for my norms and values and/or my faith in the foster family	6	7	6	**3**	5	6	4	6
20. I want to frequently do nice things with the foster family	3	4	2	4	5	5	**2**	3
21. I find it important that my biological parents honor the agreements that are made (with foster care)	**2**	5	5	6	5	5	3	4
22. I want my foster care worker to talk with me separately about how it is going in the foster family	5	3	4	**9**	4	3	**6**	4
23. I find it important to really do my best not to disappoint my foster parents	**6**	2	5	3	3	3	**7**	3
24. I find it important that my biological parents support me in everything I do	2	**7**	2	2	**1**	5	5	5
25. I find it important to do activities (like hobbies or doing nice things with friends), because it helps me to forget my past for a moment	**8**	3	4	3	4	5	5	3
26. I want my foster parents to prepare me to stand on my own two feet	6	7	6	6	8	6	**2**	6
27. I want to understand what happened in the past at my parents’ home	5	3	7	5	7	**4**	7	**5**
28. I find it important to feel secure that I can stay in my foster family until I am old enough to live on my own	6	4	**3**	**8**	**6**	9	**3**	7
29. I find it important that I can get (professional) help to process difficult things from my past when I need to	8	5	8	5	7	**5**	6	7
30. I find it important that I can always contact my guardian when I need to	**4**	2	3	**8**	4	4	4	5
31. I find it important that my foster parents take my personal boundaries into account and do not ask too much of me	6	4	6	6	5	7	9	6
32. I want to feel at home when I am with my biological parents	2	**7**	3	2	2	**8**	**5**	2
33. I find it important that my guardian or foster care worker completely explains when a choice is being made for me	5	5	5	**8**	**6**	7	8	**5**
34. Having an object from my biological parents’ home is important because it gives me support	1	1	2	1	2	**1**	3	2
35. I find it important to tell my biological parents everything about what I am experiencing in my foster family	1	2	1	3	1	4	4	1
36. I want to be able to do hobbies and activities in my foster family that suit me	4	6	6	6	**6**	**7**	**2**	**4**
37. I find it important to be able to completely be myself in my foster family	9	8	7	8	9	8	**6**	9
38. I want to be able to process missing my parents	2	4	6	2	3	1	**5**	**8**
39. I want to have friends that I can always go to when things get difficult or when I don’t feel at ease with myself	5	5	7	4	**6**	7	**9**	7
40. I find it important that my foster parents help me to understand how my past can influence my behavior or reactions	9	5	5	9	**8**	**3**	6	5
41. I want to consciously keep distance from my foster family and I don’t want to let them close to me	1	1	2	1	1	1	**7**	1
42. I find it important that my foster parents see me as their son/daughter	7	8	**1**	**3**	**5**	**2**	**1**	**9**
43. I want to be able to decide on my own whether to break the contact with my biological parents	**4**	6	**9**	7	7	7	5	5
44. I find it very important to finish school and to get an education	6	**9**	7	**5**	8	8	9	8
45. I want to be seen as more than just a foster child	6	6	**4**	7	5	**4**	6	**8**
% Explained variance	19	14	15	13	16	16	8	14

Each number indicates the score of the statement within the weighted average Q-sort of the factors (factors 1–4 for the SA group and factors A–D for the No SA group). A score of 1 reflects the most unimportant statement for that factor, and a score of 9 the most important statement. Bold values represent significantly distinguishing statements at *p* ≤ 0.05 within the SA or the No SA group

**Fig. 1:**
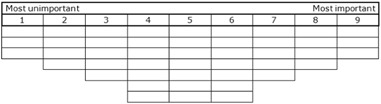
Sorting grid of the Q sort

After filling out the demographic questionnaire, participants were first asked to read through all the statements and to sort them into three piles to ease the Q sorting: important for me, unimportant for me or neutral/not applicable. Secondly, they sorted all statements in a fixed grid according to how important or unimportant each statement was for them during their foster care period. This grid ranged from most unimportant (1) to most important (9), with fewer cards fitting underneath the far sides and more cards fitting underneath the neutral middle (see Fig. 1). After sorting the statements, participants were invited to fine-tune their Q sort and, if they wished so, to elaborate on statements they found particularly important or unimportant.

### Data Analyses

Each of the Q sorts of the SA group and the No SA group were entered into the software program PQ Method (Schmolck 2002) and analyzed separately. We applied Principal Component factor analyses with Varimax rotation, which is commonly used in Q methodological studies (Brown 1980). In a by-person factor analysis, participants’ sorting of statements are subject to a factor analysis. Hence, factors are extracted based on correlations between participants, instead of correlations between items, as would be done in a conventional factor analysis. Several factor solutions were explored for both groups based on eigenvalues, explained variance, correlations between factors, number of participants significantly loading on a factor, and interpretative value (Watts and Stenner 2012). Each factor of the final solution was converted back to a weighted average Q sort of the participants loading significantly on that factor, making it easier to interpret the configuration of the statements eliciting the views within each factor. Additionally, the software program indicated consensus statements across the factors as well as which statements significantly differed between the factors, the so-called ‘distinguishing statements’. Elaborations from the participants on their Q sort supplemented the interpretation of the factors.

To examine if and how the needs of participants in the SA group and the No SA group differed, the average Q sorts of the factor solutions of the two groups were subsequently entered into the PQ Method software. This ‘second order factor analysis’ revealed if the average Q sorts of the two groups loaded on similar or different second order factors. In addition, the questionnaire data of the two groups were compared using regression analysis.

## Results

A four-factor solution was chosen for both groups of participants, with a significant loading on one factor for all participants in the SA group. Within the No SA group, four of the 25 participants did not associate with one particular factor, and were hence excluded from further analysis. The correlation between factors was relatively low; for the SA group correlation between factors ranged from 0.26 to 0.54, and for the No SA group from 0.02 to 0.57. Despite some overlap, the analyses indicated different perspectives on experienced psychosocial needs, both within and across both groups.

The weighted average Q sorts, which provide an overview of the typical arrangement of statements (from 1 to 9) for each factor, are provided in Table 1. Significantly distinguishing statements within the SA or the No SA group are indicated in bold. This is followed by a short narration of each factor based on the most important/unimportant statements and distinguishing statements, supplemented with the interview data. Factors 1–4 refer to the SA group, and factors A–D refer to the No SA group. The position of statements within each factor is indicated in the narration, i.e. when statement 2 is deemed most important (position 9), this is referred to as ‘#2/9’.

### SA Factor 1: Help Me Process My Past (*n* = 5)

The features of this factor indicated a quite ambivalent perspective while in foster care, focused mainly around Maslow’s (1943) safety and belongingness needs. On the one hand, participants in this factor found processing the past with help from foster parents and professionals important (#29/8, #40/9), while at the same time, they disclosed a need for space to deal with the past themselves (#10/7, #25/8). Making autonomous decisions regarding their lives was important (#4/8, #18/9, #37/9), but also to emotionally connect with foster parents in order to feel safe and accepted (#11/8, #41/1, #42/7). Furthermore, they found it important not to disappoint their foster parents (#23/6). Compared with other factors in the SA group, contact with birth parents seemed insignificant for these participants (#2/3, #21/2, #32/1, #38/2, #34/1, #43/4).

### SA Factor 2: I Need to Work Towards the Future (*n* = 2)

In many ways, this factor communicated a strong need oriented towards self-actualization and shaping their personal future while in foster care (#14/8, #18/9, #23/2, #37/8, #44/9). Regarding their belongingness needs, youth appreciated the support from both their foster parents and birth parents (#14/8, #24/7). They needed to feel welcomed as family member in the foster family (#11/9, #17/7, #41/1, #42/8). Moreover, they tried to establish a balance in the contact with their birth parents, who continued to play an important role in their lives (#24/7; #32/7), without them being too involved (#34/1, #35/2). These youth felt no need to dwell on the past (#3/2, #8/8, #10/1), and the need for professionals’ involvement was limited and was only present when struggles arose (#16/6, #22/3, #30/2).

### SA Factor 3: I Want to Decide About My Own Life (*n* = 5)

This factor revealed a clear need for autonomy and a limited sense of belonging (#9/8, #18/8, #29/8, #43/9). Despite a need for a safe and close relationship to the foster parents (#11/9, #41/1), these youth did not want them or professionals to be too involved in their lives (#14/5, #20/2), and being a member of the foster family was not considered as important (#28/3, #42/1, #45/4). These participants also seemed to have a more problematic relationship with their birth parents (#24/2, #34/2, #35/1), and they found it important to prevent adopting negative behavior from them (#5/9, #29/8). Several of the statement scores underpinned their self-esteem needs, which to them meant standing on their own feet. If support was needed, someone neutral was preferred (#7/6, #16/8).

### SA Factor 4: Professionals and Foster Parents Need to Help Me (*n* = 3)

The fourth factor seemed to be oriented towards the role of professionals and foster parents, disclosing rather instrumental expectations regarding their safety needs. Firstly, these youth needed their foster care workers to take their job seriously, indicating that professionals should be available (#30/8), communicate openly about care processes (#13/9, #33/8), and be a mediator between them and their foster families (#22/9). Furthermore, they needed foster parents to help them understand the influence of their past (#40/9). Despite wanting close and long-lasting relationships (#28/8, #41/1), a warm and loving home seemed less important (#11/4, #19/3, #42/3). Wanting help from their foster parents without an emotional bond was the most striking feature of these youth. Friends or other neutral persons did not need to assist youth in processing the past (#3/1, #7/2), and contact with birth parents was not important (#24/2, #32/2, #34/1, #38/2). Their focus was on learning about themselves and how their past influenced them (#22/9, #37/8, #40/9).

### No SA Factor A: I Want to Become an Independent Individual (*n* = 9)

Maslow’s (1943) self-esteem and self-actualization needs were the most important for this group of participants, who wanted to experience growth and independency (#8/7, #18/9, #26/8, #28/6, #36/6, #37/9, #44/8). Growth for these youth also meant preventing negative consequences of the past (#5/9, #40/8). To these youth, foster parents were their primary source of support for their growth needs (#26/8, #40/8, #41/1). This complied with their need for honest and caring foster parents (#1/8, #11/7, #41/1); however, it was less important for them to be regarded as their foster parents’ son or daughter (#42/5). This shows a sense of belonging that was not characterized by familial relationships. Support from professionals and others outside the foster family seemed less prominent for this group (#16/4, #17/2, #33/6, #39/6). In addition, support from and contact with birth parents was considered as unimportant (#2/2, #24/1, #32/2, #34/2, #35/1).

### No SA Factor B: I Need Support From Both My Families (*n* = 7)

The importance of support from and belonging to both the birth and foster family seemed to characterize Factor B. They needed a safe and stable situation in both families in order to work on themselves and towards their future (#11/9, #28/9, #37/8, #41/1, #44/8). Furthermore, they felt a need for support in making independent decisions (#1/6, #18/9, #36/7). They seemed to have a need to position themselves as a ‘foster child’ (#42/2, #45/4), because of the important role birth parents played in their lives (#2/8, #9/2, #32/8, #34/1, #38/1). Moreover, these youth seemed less focused on past experiences (#3/2, #12/2, #27/4, #29/5, #40/3).

### No SA Factor C: I Only Rely on My Friends and Myself (*n* = 3)

This factor portrayed youth who needed to keep foster parents at an emotional distance (#1/2, #11/3, #12/6, #14/1, #20/2, #26/2, #28/3, #31/9, #41/7, #42/1) and to have friends and professionals meet their needs (#3/8, #16/8, #22/6, #33/8, #39/9). These youth only felt a sense of belonging towards their friends, nonetheless, processing the past was considered a private matter (#10/7). These youth felt a need to make their own decisions about their future (#14/1, #18/8, #44/9) and to understand decisions made by professionals (#33/8). Statements concerning contact with their birth parents were given a rather neutral position (#2/3, #24/5, #32/5, #38/5), and they themselves wanted to decide whether and how contact should be arranged (#15/1, #21/3). The arrangement of statements seemed to communicate that these youth did not perceive the adults around them as providing them safety.

### No SA Factor D. I Am at Home in My Foster Family (*n* = 6)

Factor D revealed a perspective in which a sense of belonging and inclusion seemed to be key elements. Compared to the other factors, this factor was unique in the sense that these young adults had a strong need for being included as a true member of the foster family (#42/9, #45/8). Warmth, trustworthiness, honesty and acceptance seemed to be crucial factors for establishing close relationships with the foster family (#1/8, #11/9, #13/7#, #37/9, #41/1). These features may be seen in relation to them needing foster parents to help them make decisions and support their (self-actualized) future (#1/8, #18/6). Understanding and processing the past was relatively unimportant for these youth (#5,2, #27,5, #40,5). Furthermore, these youth did not express a need for a close relationship with their birth parents (#2/3, #32/2, #34/2, #35/1, #38/8), and they preferred talking to a neutral person if they needed someone to talk to (#7/7). In the interviews, three participants indicated that their birth parents had passed away.

### Second Order Factor Analysis

To further examine the similarities and differences between the two groups of participants, the eight factors were entered into PQ Method, and a second order analysis was run. This procedure resulted in four new factors, revealing how the factors from the two groups comply with each other (see Table 2). Second order factor (SOF) 1 and SOF 3 were formed by participants from both the SA and the No SA group, explaining 31% and 19% of the variance respectively. However, SOF 2 was solely based on the No SA group (corresponding with factor C, explaining 14% of the variance) and SOF 4 was solely based on the SA group (corresponding with Factor 4, explaining 16% of the variance).

**Table 2:** Significant factor loadings of the second order factor (SOF) analysis

	SOF 1	SOF 2	SOF 3	SOF 4
1. Help me process my past	0.67			
2. I need to work towards the future			0.78	
3. I want to decide about my own life	0.77			
4. Professionals and foster parents need to help me				0.91
A. I want to become an independent individual	0.83			
B. I need support from both my families			0.86	
C. I only rely on my friends and myself		0.96		
D. I am at home in my foster family	0.72			
% Explained variance	31	14	19	16

Lastly, in order to assess other differences between the SA group and the No SA group, their questionnaire data were compared (see Table 3). The SA group experienced significantly more placements compared to the No SA group: 3.20 placements on average compared to 1.93 placements. Moreover, the longest placement of the SA group was on average one year shorter, and they had on average two more adverse experiences prior to foster care compared to the No SA group.

**Table 3:** Means of the questionnaire data of the SA and No SA group

	SA group (*n* = 15; 2 males)		No SA group (*n* = 29; 7 males)		
	*M*	*SD*	*M*	*SD*	*p* values^a^
Age	21.67	2.87	20.59	2.96	0.25
Number of placements^b^	3.20	2.11	1.93	1.25	0.04
Longest placement^b^	7.23	5.71	8.18	5.41	<0.001
Age of first placement	7.49	4.10	8.80	4.94	0.38
ACEs prior to first placement^b^	5.53	2.70	3.52	2.52	0.03
BSI	38.47	39.07	33.55	32.61	0.66
DTS	38.53	36.06	32.10	23.47	0.09

The composition of males and females was not different between the SA and No SA group (*p* = 0.41)

^a^ *p* Values calculated with regression analysis. The significance level was set at *p* ≤ 0.05

^b^ Age of first placement was added as co-variate

## Discussion

The findings show that children in foster care differ with regard to what they consider as (most) important needs, and how these differences are related to a history of sexual abuse. The Q sort revealed eight groups of youth with distinct needs, emphasizing Maslow’s (1943) psychosocial needs (safety, belonging, self-esteem and self-actualization) differently.

Regarding our first research question, ‘what are the needs of youth according to their own views’, there are two important contrasting themes within the eight factors. The first theme is the need for help versus the need for independence. The need for help indicated by some groups highlights that children need the support of their environment to learn, grow and cope with distress (Maslow 1943; Schofield and Beek 2009). The need for help and support from others goes beyond a simple need to belong, but rather shows how close relationships impact all levels of Maslow’s need hierarchy. The need for help might be especially prominent for children in foster care, due to their tumultuous histories and the problems stemming from this, such as mental health issues (Greeson et al. 2011; Oswald et al. 2010). The effectiveness of interventions targeting mental health issues increases when youth perceive them as needed (King et al. 2014).

The need for independence, in terms of Maslow (1943) a self-esteem need, is perhaps not an unexpected finding for care leavers. They often have to be self-reliant and independent sooner than most other youth (Courtney et al. 2011). However, youth indicating the need to be independent without any form of support risk alienating themselves from their network, which might result in a lack of valuable material and emotional support (e.g., Hiles et al. 2014). Avoiding a sense of belonging might result from experiencing rejection or abandonment from adults (Skoog et al. 2015), or from dissatisfaction with the autonomy they receive (see e.g., Geenen and Powers 2007; Tatlow-Golden and McElvaney 2015). Youth who balance the need to belonging and their self-esteem needs, will possibly be better equipped to deal with everyday stressors. They learn the skills to independently handle everyday life, but also have their network as a source of support (e.g., Hiles et al. 2014; Leve et al. 2009). In line with this, supportive interpersonal relationships have been linked to resilience of youth in foster care (Leve et al. 2009).

Another contrast that emerges from the data is that youth either have a more retrospective or a more prospective orientation. Some youth in this study express the need to process their past. Actively working out past experiences satisfies youth’s need for safety, and can reduce the negative impact of previous adversities on both developmental and socio-emotional outcomes (Bruskas 2008). However, not all youth who wanted to process the past did so with the help of their foster parents. Parenting traumatized children therefore means finding a balance between becoming emotionally close enough to provide support, but not so close that it becomes overwhelming for the children and for themselves (Ironside 2004). In the Netherlands, foster parents are prepared for this difficult task by receiving training prior to becoming foster parents and are supported by foster care professionals during a placement (Pleegzorg Nederland 2017).

Contrary to youth with a more retrospective perspective, other youth indicated that they prioritized self-actualization needs and seemed very focused on their future. This meant finishing their education and learning how to live independently, all with the support of their foster parents. School achievement is an important protective factor for psychosocial problems among foster care alumni (Forsman et al. 2016), which is often achieved with the additional support from foster parents, schools and the community (Hiles et al. 2014; Morton 2016). However, for many foster care alumni it appears difficult to find stability with regard to their living situation, education, and supportive relationships that satisfy their need to belong (e.g., Geenen and Powers 2007; Hiles et al. 2014). Furthermore, purely focusing on the future might for some youth be an avoidant coping strategy aimed at reducing the emotional reactions triggered by memories of the past (Hanney and Kozlowska 2002). Hence, in order to help children to succeed in school and in life, it is crucial to identify those who have a healthy future perspective and those who employ such a future orientation as an avoidant and unhealthy strategy and help them find more useful strategies to meet their need for safety.

Regarding our second question, the results indicate that a self-reported history of sexual abuse has an influence on the needs of some, but not all youth. In line with previous research, children in foster care with a history of sexual abuse were more likely to have unstable foster care experiences and more adverse childhood experiences prior to care (Edmond et al. 2002; Pollock and Farmer 2005). However, our study suggest that most needs of the youth reporting a history of sexual abuse overlap with the needs of youth without a history of sexual abuse.

Nonetheless, one perspective was unique for the group of youth who reported being sexually abused. Youth with this perspective indicated not wanting an emotional connection to foster parents and professionals, but instead an instrumental, professional relationship characterized by a limited sense of belonging. This emotional distance and the high care needs of sexually abused children are also found in other studies (Farmer and Pollock 2003; Hardwick 2005). These children may have difficulties establishing close relationships because of the specific nature of sexual abuse. Perpetrators abuse their relationship with children and their power over children to initiate the sexual abuse (Putnam 2003). In contrast with other adverse experiences, this involves grooming children in order to gain access to them, to have children comply with the abuse and to keep them from talking about the abuse (Craven et al. 2006). As theorized by Maslow (1943), when safety needs have not been met, the need for belongingness cannot be optimally satisfied. Youth first need to feel safe with their caregivers and feel confident that their relationship will be free of abuse, before they can form a close bond.

### Strengths and Limitations

A strength of Q methodology is that it allowed us to capture diverse perspectives of youth themselves. By incorporating all four psychological needs of Maslow’s hierarchy, the youth perspectives covered a broad range of needs and illustrated the interrelatedness of these needs. In addition, the participants had the opportunity to enrich the data through explaining their sorting in a short interview.

The participants provided important insights by revealing their viewpoints through the Q sorting procedure. That said, other viewpoints may also exist ‘out there’, as other youth in foster care may hold different viewpoints. The participants varied in age, foster care experiences and current living situation, but more women than men participated, and more youth without a history of sexual abuse. Although the questionnaires showed that the current levels of trauma symptoms and psychological problems varied from low to high among the participants, perhaps youth with more serious problems did not participate. Furthermore, while the sample is of a good size for a Q-methodological study (Watts and Stenner 2012), the relatively small size and the purposeful sampling strategy limit the generalizability of the results.

A last limitation is the use of self-reports to acquire information about foster care experiences and sexual abuse. This method reveals the experiences of the participants, which aligns with the qualitative nature of this study, but is not without shortcomings. People can forget the numerous housing experiences because they were still young at that time. Moreover, people can forget or suppress sexual abuse and thus under-report this in questionnaires (Wilsnack et al. 2002). Research shows that especially less severe forms of sexual abuse are inconsistently reported (Langeland et al. 2015).

### Research Implications

This study has shown that youth in foster care find both deficiency and growth needs important. Follow-up studies can assess if meeting the needs of youth leads to greater well-being, for example by designing or evaluating a foster parent training program that addresses youth’s needs by measuring the well-being of youth on domains such as school accomplishments, quality of relationships, coping and identity. Moreover, research could focus on how and by whom these needs are met, and if the various people involved in the children’s life agree on what needs are most important. Large-scale longitudinal studies are necessary for this, preferably following a group of children throughout their care experiences. Such studies should also take abuse history into account, since this study has shown that the needs of some youth with a history of sexual abuse seem to differ from youth without a history of sexual abuse.

The diverse needs of children in family foster care also resonate with current practices in the Netherlands. Foster parents and foster care workers have the difficult task to both stimulate youth’s preparation for the future, and also promote processing past experiences. The preparation for the future not only includes education and independent living skills, but also permanency of relationships (Leve et al. 2009). This underpins the importance of the possibility to extend foster care beyond the age of 18, as is already policy within the Netherlands and other Western countries (e.g., Government United Kingdom 2014; Nederlandse Overheid 2015; Peters 2012). Furthermore, since sexual abuse affects the needs of youth, the current focus on trauma-informed care may indeed be helpful in foster care (Fratto 2016). Lastly, this study has shown that youth and young adults are capable of indicating their most important needs with the Q sort tool. Foster parents and foster care workers need to take the needs of children into consideration when making a care plan, particularly when certain difficulties arise. If verbally expressing their needs is difficult for children, further research can determine whether foster parents and care professionals could use a Q sort as a non-verbal tool to accurately determine the needs of the children and help children with voicing their needs.
